# The role of top-down and bottom-up factors in parafoveal reading

**DOI:** 10.3389/fcogn.2025.1715617

**Published:** 2025-12-02

**Authors:** Valentina Bandiera, Silvia Primativo, Roberta Daini, Marialuisa Martelli, Lisa S. Arduino

**Affiliations:** 1 Department of Human Sciences – LUMSA University, Rome, Italy; 2 Department of Psychology, University of Milano-Bicocca, Milan, Italy; 3 Department of Psychology, Università La Sapienza Roma, Rome, Italy

**Keywords:** parafoveal processing, semantic, rapid parallel visual presentation, word recognition, reading models

## Abstract

Recently we have shown that, while reading two words presented simultaneously, one in the fovea and one in the parafovea, participants are more accurate and faster when the two words are semantically related. The present study confirmed and supported the previous results by using the same Rapid Parallel Visual Presentation Paradigm (RPVP) and by changing the relative proportions of unrelated vs. semantically related word pairs. Indeed, differently from other studies where semantically unrelated and related word pairs were equally represented (50% each), in the present study only 30% of word pairs were semantically related. Results showed again an advantage when the two words were semantically related and we interpreted these findings in terms of automaticity between lexical/sublexical units processing and semantic access.

## 1 Introduction

Readers extract information not only from the words they fixate, but also from words to the right of fixation (see Rayner, 1998, for a review). Both bottom-up (i.e., basic visual features of the stimuli, such as length, position on the visual field, etc.) and top-down factors (i.e., expectations and previous knowledge, such as lexical and semantic information) play a role in this complex process (see Risse, 2014). It has been established that especially visual orthographic features of the word to the right of fixation are extracted (e.g., Balota et al., 1985; Pollatsek et al., 1992; Altarriba et al., 2001) and this has been shown mostly by studies using the boundary paradigm (e.g., Altarriba et al., 2001; Pollatsek et al., 1992), a paradigm in which letters of a parafoveal word are initially replaced by visually similar or dissimilar letters (Rayner, 1975).

Substantial evidence supports the view that parafoveal information is extracted at various linguistic levels, including orthography (e.g., Inhoff, 1989; White, 2008), phonology (e.g., Ashby and Rayner, 2004; Miellet and Sparrow, 2004; Pollatsek et al., 1992; Rayner et al., 1995), and semantics (Rayner and Schotter, 2014; Schotter, 2013; Schotter et al., 2015; Schotter and Jia, 2016). However, for semantics in particular, controversy remains about the extent and type of information extracted from parafoveal processing (Inhoff and Rayner, 1986; White et al., 2008; Juhasz et al., 2009; Rayner et al., 2014). Eye tracking-based evidence for the extraction of parafoveal semantic information emerged from studies that used languages other than English, such as Chinese (Tsai et al., 2012; Yan et al., 2012, 2009; Zhou et al., 2013), German (e.g., Hohenstein et al., 2010; Hohenstein and Kliegl, 2014), Arabic (Hermena et al., 2021), and Italian (Rusich et al., 2020; Primativo et al., 2022). Complementary evidence showing that semantic information can be extracted from parafoveal vision, even in English, comes from electrophysiological studies (e.g., Kutas and Hillyard, 1984). Thus, both eye tracking and electrophysiological studies have provided evidence suggesting that semantic information is extracted from words in parafoveal preview. Moreover, a recent study by Pan et al. (2023) provided information about the time course of semantic integration, since they found evidence that readers integrate the semantics of the parafoveal words with their ongoing message-level representation—by as early as within 100 ms after fixating on the pre-target word.

Taken together, this body of evidence, suggest that a preview of word N + 1 affects the fixation time of word N as well (e.g., Kennedy et al., 2002; Kliegl et al., 2007; White, 2008). Kennedy has coined this effect the “parafoveal-on-foveal effect,” arguing that parafoveal-on-foveal effects reflect parallel word processing, since influences of N + 1 word properties on processing word N can only be explained if one assumes that both words are processed simultaneously.

Such an issue is crucial in terms of parallel vs. serial models of reading aloud. Indeed, results indicating a lack of semantic processing of the parafoveal word (e.g., Rayner et al., 2014) have been taken as evidence of seriality in word reading and eye movement models (Reichle et al., 2009). By contrast, the literature reporting semantic parafoveal-on-foveal effects (e.g., López-Peréz et al., 2016; Snell et al., 2018; Rusich et al., 2020; Primativo et al., 2022) indicates that words in the parafovea might be fully processed and their meanings immediately integrated. The parallel processing of two adjacent words is explained by visuo-spatial attention that is distributed across multiple words within the visual field (e.g., SWIFT; Engbert et al., 2005).

In our more recent work (Primativo et al., 2022) we adopted the same paradigm used in Rusich et al. (2020), namely the Rapid Parallel Visual Presentation Paradigm (RPVP; Snell and Grainger, 2017), founding that semantic information from the parafovea emerged before 100 ms. The paradigm consisted of the simultaneous presentation of a pair of words, one in fovea (W1) and one in parafovea (W2). In three experiments, with the same stimuli but different participants, we manipulated word frequency of W1 and W2, the semantic relatedness between the two words (present or absent) and the effect of stimulus presentation time (150, 100, 50 ms).

The paradigm allowed us to measure (1) the parafoveal preview benefit (PPE effect), in terms of the accuracy advantage in reading a parafoveal word when it is semantically related to the foveal one (2) the parafoveal-on-foveal effect (PoF effect), in terms of faster reading times of the foveal word, depending on the relatedness to the word in the parafovea.

The results showed that accuracy in reading W2 was higher when W1 and W2 were both high-frequency and semantically related. Similarly, W1 reading times were faster when both words were high-frequency and semantically related (150 ms) or when W2 was high-frequency and semantically related to the foveal word (100 ms). When the stimuli were presented for 50 ms, reading times were faster when W1 was highly frequent and semantically related to the other word. Taken together, the results from the three experiments supported the view that it is possible to extract semantic information from the parafovea and that such extraction happens very early in processing (within 100 ms) and in parallel to the processing of the foveal word. This is true especially when the cognitive load required for processing the word in the fovea is reduced, as when it consists of a high-frequency word. In order to better understand the mechanisms underlying the semantic facilitation of parafoveal reading, we manipulated list composition, by changing the proportion of semantically related and unrelated word pairs. Indeed, in the previous studies (Rusich et al., 2020; Primativo et al., 2022) the experimental lists always involved an equal number of semantically related vs. unrelated word pairs (50%), while in the present study the percentage of semantically related words was reduced to 30%.

If the role of semantic facilitation is mostly modulated by expectations, we should observe a reduced semantic effect of words in parafovea when the list contains few semantically related word pairs, compared to a condition in which participants encountered the same proportion of semantically related and unrelated stimuli. Expectations reflect proactive top-down processing. A reduction in semantic facilitation, associated with fewer semantically related word pairs, would support their role in this phenomenon. On the other hand, if words that are related are broadly activated, multiple words may be activated at once, and this activation may be graded rather than all-or-none. Such facilitation is not due to anticipatory processes, but rather to more efficient automatic integration of predictable words (Veldre and Andrews, 2015).

## 2 Method

### 2.1 Participants

The experimental protocol was approved by the local ethics committee of LUMSA University (CERS) under protocol number 16/2023. Participants were recruited from January to June 2025, and all provided written informed consent. Based on a G-Power analysis (Faul et al., 2009; power = 0.95, alpha = 0.05, effect size = 0.6), we determined a sample size of 32 participants.

All participants self-reported normal or corrected-to-normal vision and no history of dyslexia. Thirty-four participants were recruited for the study. Two of them were excluded because of low accuracy (below 30%) in one of the experimental conditions. Thus, the final sample consisted of 32 participants (mean age = 23 years; range = 19–29 years; SD = 3.03; 26 females; mean education = 15 years). All participants were naïve to the purpose of the study.

### 2.2 Stimuli

One hundred and thirty-six pairs of Italian nouns were selected from the CoLFIS database (Bertinetto et al., 2005). Written word frequency (mean = 217.2 occurrences per million, range = 25–1,469, SD = 272.4) and semantic relatedness between the two words were manipulated orthogonally. All words were four–five letters in length (mean= 4.5) and only nouns that had a frequency of at least 25 per million were included. Extremely high-frequency (H-F) words (>2.5 SD above the mean, i.e., >1,580) were excluded. The median frequency was 92; thus, words below 92 were categorized as low-frequency (L-F), and those above 92 as high-frequency (cf. Barca et al., 2002). For the purpose of the present study, we created an unbalance stimulus list composed of 30% semantically related (SR) and 70% semantically unrelated (SU) word pairs. We started from a list composed of 50% SU and 50% SR word pairs, created on the basis of the semantic relatedness judgment determined through a 7-point Likert scale questionnaire administered to 33 independent participants (mean age = 21.4 years, range = 20–23 years, SD = 0.8, 30 females) who did not take part in the main experiments. SR pairs (e.g., PANE-VINO; BREAD-WINE) received significantly higher ratings (mean = 5.8, SD = 1.2; range = 2.5–7.0) than SU pairs (e.g., RIVA-ARTE; SHORE-ART; mean = 1.7, SD = 1.2; range = 1.0–5.3). To further verify the semantic relatedness judgment, we used another method namely WEISS (Word-Embedding's Italian Semantic Space), a distributional semantic computational model based on Italian, devised by Marelli (2017). This model provides semantic relatedness estimates on the basis of cosines between vectors: the more related two words are, the closer their corresponding vectors are, and the higher the cosine of the angle between them (Marelli, 2017). On the basis of this method, we observed that the mean value in our SU starting list (50% SU and 50% SR word pairs) was 0.08 (SD = 0.08). The mean value in our SR starting list was 0.33 (SD = 0.15). To achieve the 30%-70% proportion, we added 56 pairs of unrelated words to the original list. In order to do so, we used a cut-off of 0.24; pairs of words below this cut-off were considered semantically unrelated. By doing so, our final list of words consisted of 40 SR pair of words (the same as in the original list) and 96 SU pair of words. In each list, half of the stimuli were of high-frequency and half were of low-frequency (i.e., 48 HF vs. 48 LF words in the SU list and 20 HF vs. 20 LF words in the SR list). In each pair, the first word (W1) was presented in fovea, and the second word (W2) in parafovea. Horizontally, the stimuli extended from 7.5 to 9.5 cm (min–max). The word pairs did not involve synonyms or antonyms.

### 2.3 Software and apparatus

The Experiment Builder software (SR Research Ltd., Mississauga, ON, Canada) was used for programming and running the experiments. To ensure stable fixation and precise retinal positioning of the stimuli before their presentation on the screen, eye movements were recorded using an SR Research Ltd. EyeLink 1000 Plus eye-tracker, sampling at 1,000 Hz. Participants were seated approximately 57 cm from a 27-inch LED monitor (1,366 × 768 pixels, 60 Hz), with head movements restricted by a chin rest. Vocal responses were recorded via a one-way microphone connected to an external sound card (M-track 22).

### 2.4 Experimental procedure

At the beginning of each experiment, a standard nine-point calibration and validation procedure was conducted. Each trial began with a fixation cross (subtending a visual angle of 0.5°) displayed on the left side of the screen. Once fixation was held for at least 250 ms, the cross disappeared, and a pair of words (W1 and W2) appeared for 150 ms. The fixation cross was positioned between the second and third letters of W1, corresponding to the optimal viewing position (O'Regan, 1992). The order of presentation of the pair of words was randomized across participants. W1 was displayed in the fovea and W2 in the parafovea. Words were presented in Courier New, a monospaced font ensuring equal center-to-center letter spacing. Each letter subtended 0.5° of visual angle. The total horizontal extent of the word pair ranged from 8.4° (both words four letters) to 10.5° (both words five letters). Participants were instructed to read both words aloud as quickly and accurately as possible. Each trial ended with a response screen, followed by a 3,000 ms intertrial interval. The following dependent measures were recorded: (1) Vocal response times (vRTs) to W1; (2) Accuracy in reading W1 and W2.

### 2.5 Data cleaning and statistical procedure

The main dependent measures were vRTs and accuracy in reading. We excluded from the analysis two participants due to low accuracy on W2 (below 30%). Furthermore, by visual inspection, we excluded trials containing saccades directed toward the parafoveal word (1.5%). Analyses for W2 accuracy included only trials where W1 was correctly named. vRTs were analyzed only for trials in which both W1 and W2 were correctly read. Outliers (vRTs exceeding ± 2.5 standard deviations from each participant's mean) were removed. Data were analyzed using repeated measures ANOVAs, with three within-subjects' factors: W1 frequency (W1-F, high vs. low), W2 frequency (W2-F, high vs. low), and semantic relatedness (semantically related or semantically unrelated, SR vs. SU, respectively). Significant interactions were further explored with Tukey *post-hoc* tests, and effect sizes are reported as partial eta squared (η*p*^2^).

## 3 Results

### 3.1 Accuracy in reading on W2

Mean accuracy was 94.2% for W1 (range = 79%−99%) and 66.75% for W2 (range = 32%−91%).

**Figure 1:**
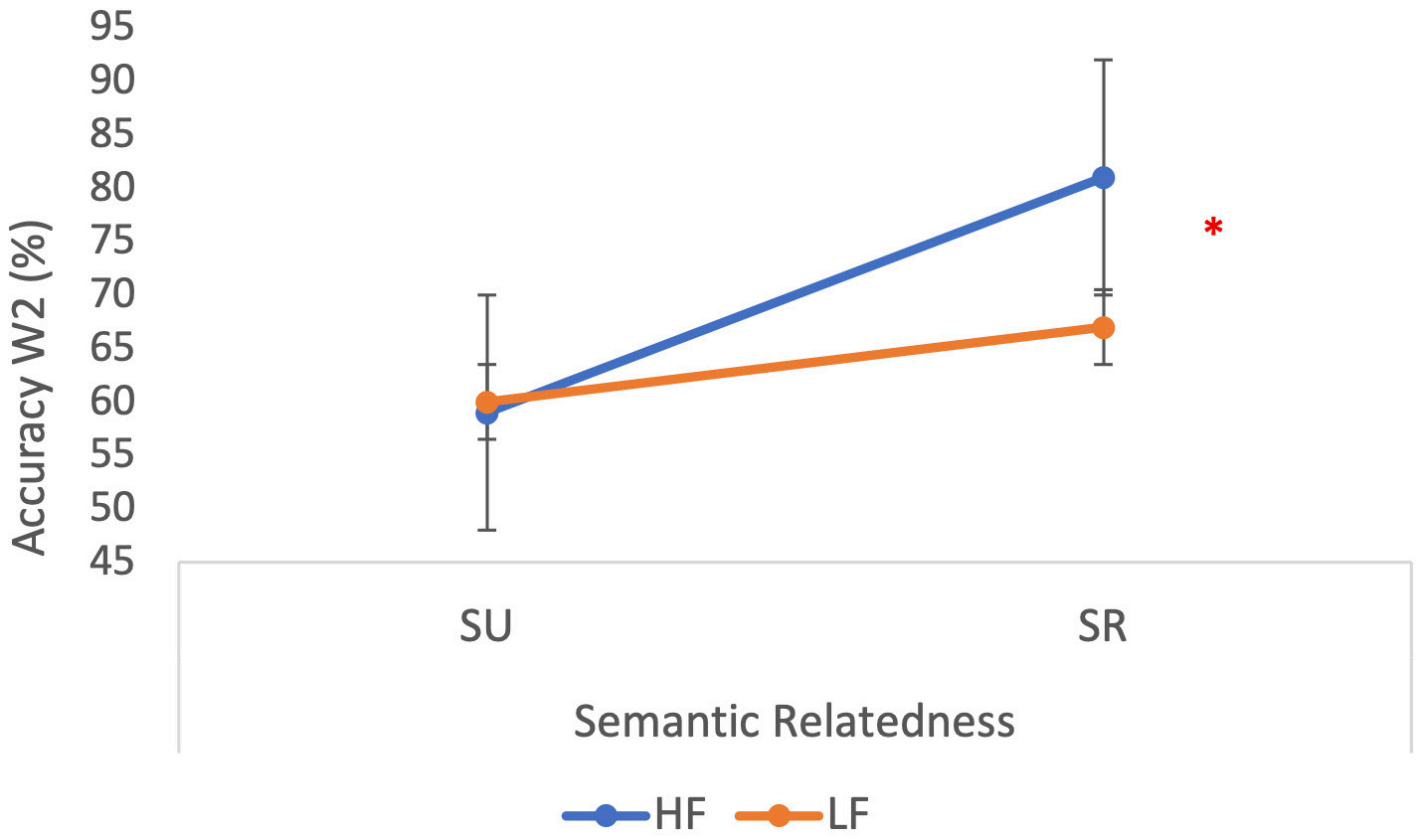
Accuracy in reading W2. Significant interaction between W2 frequency (HF, high-frequency; LF, low-frequency) and semantic relatedness (SU, semantically unrelated; SR, semantically related). The asterisk (*) indicates statistically significant differences (*p* < 0.05). The bars indicate the standard errors.

The main effect of W1 frequency was not significant (*p* = 0.07). Conversely, a significant main effect of W2 frequency (W2-F) was observed, showing higher accuracy in reading the second word when it was of high-frequency [69.8% vs. 63.7%; *F*_(1, 31)_ = 25.6, *p* < 0.001, η*p*^2^ = 0.45]. A significant main effect of semantic relation (SR) was also observed with greater accuracy when the two words were semantically related [74% vs. 59.5%; *F*_(1, 31)_ = 70.2, *p* < 0.001; η*p*^2^ = 0.70]. Furthermore, the interaction between W2-F and semantic relatedness [*F*_(1, 31)_ = 35.8, *p* < 0.001; η*p*^2^ = 0.54] was also significant. *Post-hoc* comparisons revealed that the accuracy in reading the second word was significantly higher when the second word was of high-frequency and semantically related to W1 compared to the situation in which the second word was of low frequency (80.8% vs. 67.2%; *p* < 0.001, respectively). The interaction is illustrated in Figure 1.

### 3.2 Vocal reaction times on W1

**Figure 2:**
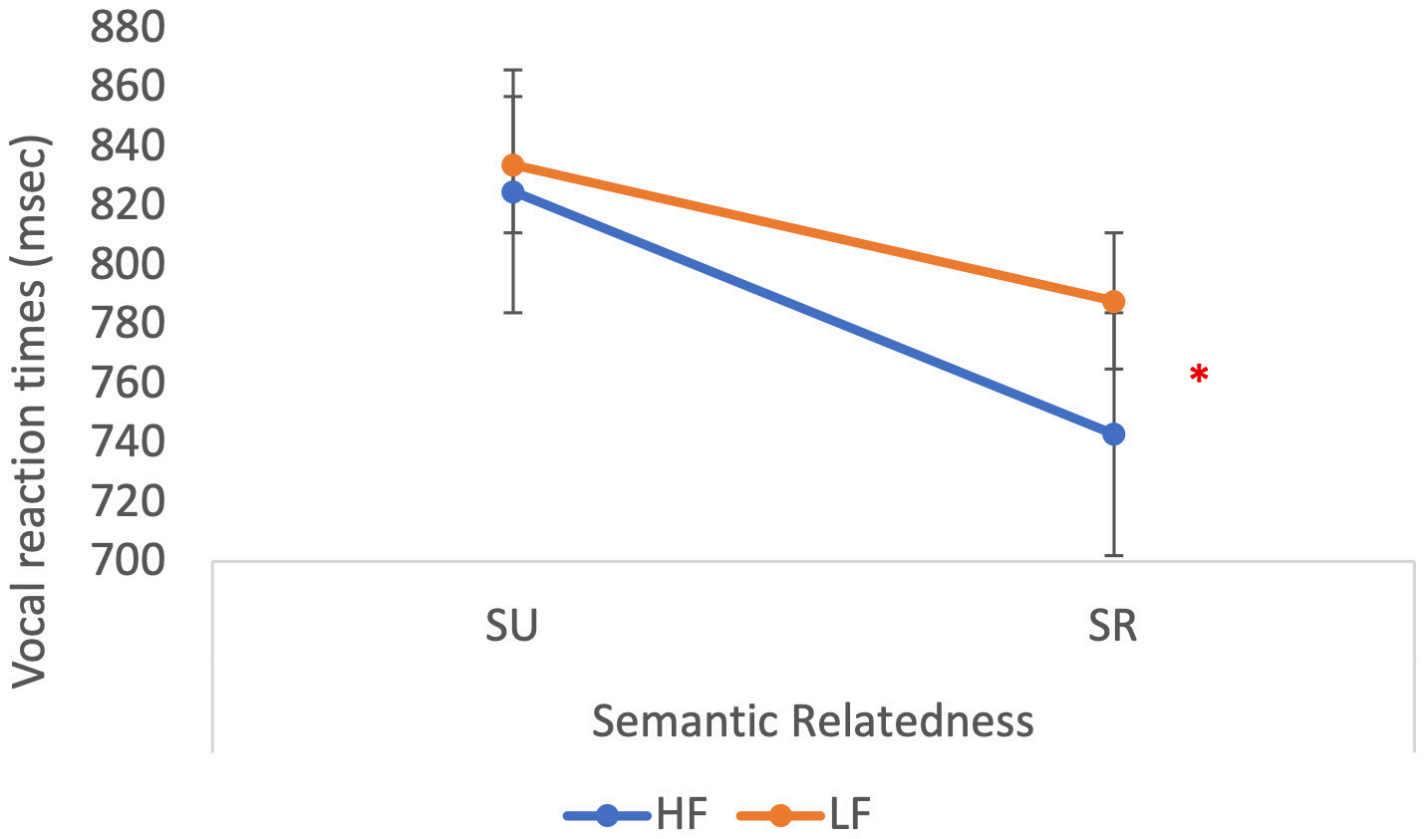
Vocal reaction times on W1. Significant interaction between W1 frequency (HF, high-frequency; LF, low-frequency) and semantic relatedness (SU, semantically unrelated; SR, semantically related). The asterisk (*) indicates statistically significant differences (*p* < 0.05). The bars indicate the standard errors.

ANOVA showed a main effect of W1-F [784 vs. 811 ms; *F*_(1, 31)_ = 9.4, *p* < 0.01; η*p*^2^ = 0.23], indicating that response time in reading the first word was faster when it was of high frequency. The main effect W2-F [784 vs. 810 ms; *F*_(1, 31)_ = 10.0, *p* < 0.01; η*p*^2^ = 0.24] was also observed, showing faster naming times for the first word when the second was of high frequency. A main effect of semantic relation was also statistically significant [*F*_(1, 31)_ = 27.6, *p* < 0.001; η*p*^2^ = 0.47], showing that W1 was named faster when the two words were semantically related (765 vs. 829 ms). A significant interaction between semantic relatedness and W1-F was found [*F*_(1, 31)_ = 7.5, *p* < 0.05; η*p*^2^ = 0.20]. *Post-hoc* comparisons revealed that the vocal reaction times on W1 were significantly faster when W1 was of high-frequency and semantically related to W2 (743 vs. 788 ms; *p* = 0.001), compared to when the first word was of low frequency. The interaction is depicted in Figure 2.

## 4 Discussion

In this study we confirmed and further supported previous results regarding a facilitation of parafoveal reading when the word in parafovea was semantically related to the word in fovea, in a Rapid Parallel Visual Presentation Paradigm (RPVP). Furthermore, the parafoveal word frequency affected the foveal word processing further supporting the view of parallel processing during reading. The parallel processing of two adjacent words is explained by visuo-spatial attention that is distributed across multiple words within the visual field. This graded allocation of attention accounts for the parafoveal-on-foveal effect (Kennedy and Pynte, 2005), and parafoveal preview effect (Inhoff and Rayner, 1986), whereby parafoveal word properties influence foveal processing.

Moreover, the present study provides a better understanding of the effect of semantic properties of parafoveal word, showing that even when the percentage of semantically related couples of words was only 30% of the total (in a list made up of semantically related and unrelated pairs of words), the semantic facilitation is still present. Such results are in line with many other studies also with different languages which demonstrated a rapid extraction of semantic representation and its effect on word recognition (Asano and Yokosawa, 2011; Hermena et al., 2021; Rami et al., 2023). In particular, Hermena et al. (2021) demonstrated parafoveal processing of orthographic, morphological, and semantic information in Arabic, a right-to-left language, using the boundary paradigm. Notably, their findings suggest that semantic processing may occur even earlier than orthographic processing, supporting the interpretation of early semantic activation in parafoveal vision.

It may be argued that even if the semantic parafoveal effect observed in free response arises from guessing, it remains relevant to natural reading, where guessing may play a role in generating the effects of lexical predictability that are reliably observed (see Staub, 2015, for a review).

However, the role of actual guessing in normal reading—unconstrained by bottom-up evidence—is limited. For example, while it is well-established that readers' eyes do skip predictable words at a relatively high rate, they do so only when they are able to obtain parafoveal visual information that is consistent with the predictable word (e.g., Balota et al., 1985), thus indicating that pre-lexical processing has taken place. Indeed, the interesting point is whether predictability effects are due to genuinely predictive or anticipatory voluntary processes based on lexical activation, or a more automatic bottom-up process. The first possibility is that a reader will sometimes maintain a specific, discrete prediction about what word is likely to come next and that words that are related to the word in fovea are broadly activated; multiple words may be activated at once, and this activation may be graded rather than all-or-none. If this is the case, however, we should have obtained a less strong effect of semantic activation in parafovea when the list contained a smaller proportion of semantically related pairs, as in the present study. But this was not the case. However, if the bottom-up activation of lexical and sublexical units connected with semantics is automatic, this would occur independently of list composition. In this case, bottom-up information refers to the lexical and sub-lexical representations derived simply from the visual input. This is exactly what we found.

Taken together, these tentative theoretical conclusions suggest that, the overall picture that emerges is as follows. Reading results in the activation of potential upcoming words. This does not seem to involve prediction of a specific word, but rather graded activation of words approximately in proportion to their cloze probability, coming from different processing units [e.g., lexical, sublexical, engrams (Marelli, 2017)] which, in turn, activate semantics.

Until recently, most experimental evidence favored a hypothesis that parafoveal processing is restricted to low-level features. However, Risse (2014) reported that the relationship between visual span, reading speed and parafoveal preview benefit are not so straight-forward, with the amplitude of the visual span impacting on the reading speed (via the modulation of the fixation durations) but not the benefits in terms of parafoveal processing.

Furthermore, Yan et al. (2009) argued that priority in parafoveal processing among various types of information vary for different writing systems. In English, prominent models posit sequential lexical activation, with semantic information accessed at a relatively late stage in the processing chain (e.g., Coltheart et al., 2001); activation of orthographic and phonological information is faster than that of semantic information (Schotter et al., 2012). Instead, Yan et al. (2009) proposed that the absence of semantic preview benefit in English should be considered tentative and not necessarily universal across languages. In particular, they argued that semantic information should be activated more rapidly in Chinese. The work on semantic preview benefit in Chinese has inspired a series of studies to re-explore the topic in alphabetic writing systems. In general, orthographic depth of an alphabetic writing system (i.e., the level of transparency in terms of grapheme-to-phoneme correspondence) plays an important role in lexical access. Under such a theoretical framework, Laubrock and Hohenstein (2012) argued that, as compared to English, a shallower orthographic depth in German leads to faster phonological decoding, which in turn facilitates access to semantics. This makes it possible for German or Italian readers to extract useful semantic knowledge from parafoveal words during the short fixation periods (see also Hohenstein and Kliegl, 2014; Rusich et al., 2020; Primativo et al., 2022). Moreover, electrophysiological evidence suggests that canonical visual N400 congruity effects can be elicited by semantically incongruent parafoveal previews in highly transparent Spanish. More recently, Wang et al. (2016) reported the first evidence for cross language semantic preview benefit among Korean-Chinese bilinguals. Together with benefits from cognate previews, they also found preview benefit from semantically related non-cognate words without phonological overlap. Yan et al. (2019) reported a standard semantic preview benefit during monolingual reading among native Korean adults.

In conclusion, this study suggests that semantic facilitation in parafoveal reading is not strongly influenced by expectations and, presumably, could be due to the fast automatic activation of semantic representations more than to top-down processes.

## Data Availability

The raw data supporting the conclusions of this article are freely available at the following link: https://osf.io/cxrq5?view_only=69bea4ccfecc4aafb21950a7d8932565.
